# Tumour Initiation: a Discussion on Evidence for a “Load-Trigger” Mechanism

**DOI:** 10.1007/s12013-019-00888-z

**Published:** 2019-10-09

**Authors:** John J. Evans, Maan M. Alkaisi, Peter H. Sykes

**Affiliations:** 1 Department of Obstetrics and Gynaecology, University of Otago Christchurch, Christchurch, New Zealand; 2 MacDiarmid Institute of Advanced Materials and Nanotechnology, Christchurch, New Zealand; 3 Department of Electrical and Computer Engineering, University of Canterbury, Christchurch, New Zealand

**Keywords:** Tumourigenesis, Cancer initiation, Collagen, Extracellular matrix, Mechanical forces, Biophysical environment

## Abstract

Appropriate mechanical forces on cells are vital for normal cell behaviour and this review discusses the possibility that tumour initiation depends partly on the disruption of the normal physical architecture of the extracellular matrix (ECM) around a cell. The alterations that occur thence promote oncogene expression. Some questions, that are not answered with certainty by current consensus mechanisms of tumourigenesis, are elegantly explained by the triggering of tumours being a property of the physical characteristics of the ECM, which is operative following loading of the tumour initiation process with a relevant gene variant. Clinical observations are consistent with this alternative hypothesis which is derived from studies that have, together, accumulated an extensive variety of data incorporating biochemical, genetic and clinical findings. Thus, this review provides support for the view that the ECM may have an executive function in induction of a tumour. Overall, reported observations suggest that either restoring an ECM associated with homeostasis or targeting the related signal transduction mechanisms may possibly be utilised to modify or control the early progression of cancers. The review provides a coherent template for discussing the notion, in the context of contemporary knowledge, that tumourigenesis is an alliance of biochemistry, genetics and biophysics, in which the physical architecture of the ECM may be a fundamental component. For more definitive clarification of the concept there needs to be a phalanx of experiments conceived around direct questions that are raised by this paper.

## Introduction

There have been many reports on the physical characteristics of cancer cells as they pass through the epithelial–mesenchymal transition and also the necessity of changes in the properties of the extracellular matrix (ECM) associated with that process and with cancer expansion [1–5]. In contrast, the role of the ECM in the very early development of a malignant tumour is a poorly researched area. We suggest that a change in the physical micro- and nano-environment enables the expression of oncogenes in tumourigenesis.

Here we discuss an early process that results in development of a tumour. The ECM changes to a state that no longer possesses biophysical and biochemical properties that control for healthy cell behaviour. Then genetic variations that have occurred can now express associated aberrant activity. Here we use the word ‘trigger’ to describe the effect of disruption of the ECM and loss of homeostasis that is the immediate chronological precursor of initiation of a malign tumour. For the ECM to have this effect there must be accessible genetic dysfunction, a state we term ‘loading’. The consequence of ‘triggering the loaded pistol’ is development of a clonal tumour.

The matter of cancer cure has been of concern for millennia. There have been numerous targeted methodologies proposed. Most recently there has been a huge effort expended on identification and quantitation of gene mutations that together might result in uncontrolled proliferation [6, 7]. Latterly, also, the release and enhancement of the immunological system, a revisiting of an old concept, is proving encouraging. There has been an underlying assumption that these efforts to improve treatment methodologies will themselves explain the initiation of a tumour. However it is possible that the triggering of cancer growth occurs via a different or modified mechanism from that which promotes cancer spread.

This review extends the idea that potential tumour initiation genesis is held at bay until a permissive environment develops [8]. Versions of schema that explain tumourigenesis by invoking a permissive environment more recently include the development of a variety of genetic-escape mechanisms and thence involvement of well-studied pathways, such as those involving integrins [9] or tumour suppressor genes [10]. Other hypotheses suggest that genetic instability is induced, for example by viral infection [11]. These all involve numerous steps with complex processes involving unidentified inducers of pathway activation followed by secretion of factors from the stromal cells of the ECM. However, unequivocal direct evidence for these processes in very early tumour initiation is absent and details are poorly identified. We suggest that simpler schema for tumourigenesis based on an understanding of the biophysics of the ECM [12] are sufficient to provide the basic mechanism.

This review suggests that the biophysics of a normal ECM inhibits expression of an oncogenic mutation, but the tumour phenotype is permitted when disruption of the ECM integrity has occurred. Here we will integrate evidence that is consistent with this hypothesis from several approaches, including experimental alteration of the in vitro physical micro- and nano-environment, orthotopic transplants, clinical and laboratory observation of mutations of ECM-related genes, external physical trauma and scar formation, and disruption of the ECM by aging. This review gathers evidence for the proposal from a wide range of subdisciplines—biochemistry, stereochemistry, mathematical modelling, epidemiology, cytoplasmic signalling pathways, epigenetics, mechanics, genetics, transduction processes and so on, and it networks a wide range of scales to tumourigenesis and thereby provides a coherent narrative for the manner in which mechanotransduction is fundamental to early tumour development (via oncogene expression). The importance of these considerations lies in their potential to address the discordances of many current hypotheses around tumour initiation. The review ends with illustrations of the possibility of reverting tumour cells to a normal phenotype by restoring healthy homeostasis conditions in the ECM.

## Empirical Data and Current Speculation

In spite of the research community having spent billions of dollars and committing a mammoth number of work-hours there remain some lingering and fundamental questions regarding cancer growth. Progress has been modest and is partly limited by lack of understanding of the pathways related to the trigger of tumourigenesis. One type of conundrum is illustrated by germline mutations such as those of BRCA. Their presence foretells the appearance of breast cancer in 45–60% of women by the age 70 [13]; thus a substantial number of the women do *not* get cancer, and those that do have it only in restricted parts of the body, which indicates that a BRCA mutation is not expressed except within a relevant biological/biophysical environment. Other questions revolve around the observation that cancer is usually a disease of old age [14]. Many consensus explanations are based on the proposition that a threshold number of mutations must be acquired and be effective (such as being unrepaired or being resistant to immunological responses) before oncogenic activity is manifest. However while noting that mutations occur mainly at the time of cell division (e.g. [15–17]), which is most frequent when tissues are developing early in life, it has been observed that accumulation of mutations or accumulation of gene disruptions do not exhibit the same time profiles as cancer incidence [18, 19]. Further, stem cell populations, which are associated with potential entrenchment of mutations, are highest in the early postnatal period, and conversely stem cell divisions are most frequent prior to our late teenage years [19], again exhibiting patterns widely different from the profile of cancer incidence. Therefore it seems unlikely that the accumulation of mutations can be the prime factor in cancer appearance [19]. This conclusion is in agreement with mathematical models that estimated fitness of single cells and subsequent clonal expansion mutation rates, having taken cognisance of the balance between drift versus selection and stabilising versus directional processes [19, 20] and for which inclusion of other parameters such as immunological responses were not necessary.

## Mechanical Forces

The knowledge that mechanical forces alter cell behaviour, has been extant in communities over the ages, including the Neanderthals, the ancient Greeks, Romans and Indians [21, 22]. That effects of forces occur at the cellular level was simply demonstrated on the space station where cultured cells in microgravity exhibited a difference in expression of over 15% of the 100,000 genes studied compared with cells cultured at normal gravity. Importantly in this discussion of tissue modification by forces, is the observation that those changes were consistently in genes associated with the cytoskeleton, such as, actin, fibrils, tubules and so on in a range of cancer cell types [23–25]. The importance of this association lies in the well-known mantra that echoes the tight relationship between structure of cells and tissues and their functioning. There are numerous publications reporting the connection between cell growth and division with the morphology of cells [26–28]. Thus an alteration of cytoskeletal arrangement induced by mechanical forces will logically modify function and behaviour of the cells. With particular relevance to the understanding of cancer proliferation, control by mechanical forces and cell shaping includes that of the cell cycle [29].

There are now numerous publications at the cell level [30] on the effects of a variety of types of forces (e.g. tensile, contractile, shear and others) on cells from a range of tissues e.g. bone [31], brain [32], liver [33], ovary [34], muscle [35], skin [36] and other organ tissues. There is supporting evidence being steadily reported [12, 37–39] that cancer cells are similarly responsive to mechanical forces. Indeed, evidence that physical exercise has a noticeable effect on cancer development is accumulating from a variety of studies [40, 41].

## Bio-physics and Cancer

If the biophysics of the environment controls cell behaviour then alteration of the environment will induce different cell behaviours.

**Fig. 1:**
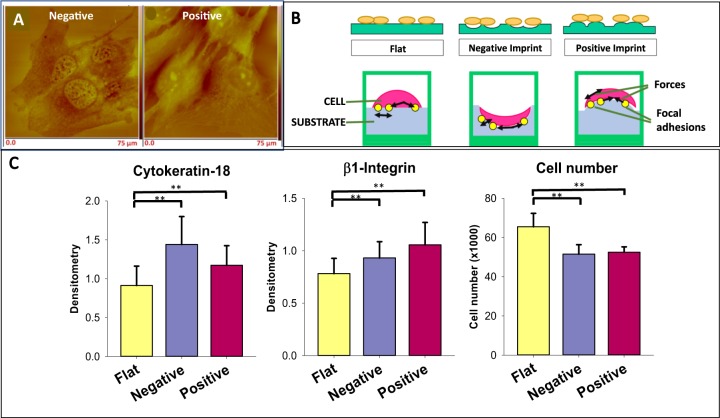
Physical topography of the substrate of cell culture regulates cell function independent of genotype and growth factors. **a** Atomic force microscope images of negative (concave) (left) and positive (convex) (right) bioimprinted replication of cells (nasal chondrocytes) prepared in polystyrene. Membrane pores are imaged as protrusions in the negative bioimprint and as concave pores in the positive bioimprint [44] (with permission of IOP Publishing). **b** Top: a cartoon of cells on flat, negative aspect and positive aspect bioimprints. Bottom: a cartoon of cells attached at focal adhesion points (yellow dot) to substrate. **c** The effect of topography (flat, yellow; negative, blue; positive, burgundy) on Ishikawa cancer cells is illustrated for cytokeratin-18 expression (left), β1-integrin expression (centre) and cell number (right) following 60 h culture [46] (with permission of Dove Medical Press)

An example of modulation of cell behaviour by altering the environment but *without* growth factor addition or gene modification is the observation [42] that the phenotype expressed by MCF-10A cells was dependent on collagen levels in culture media, and was manifest as either (i) single cells, (ii) acinar structures with single cell-to-cell extensions, or (iii) acinar structures with two cell-to-cell attachments, which provide potential to form a community of cells linked by inter-cell communications. In another study it was observed [38] that the appropriate *physical* microenvironment is able to promote the cancer stem cell phenotype. Cells of the murine B16 melanoma cell lines were cultured on a variety of circular patterns. Cells on the perimeter islands of a culture exhibited morphology, proliferation and integrin expression that was sensitive to pattern sizes. Further, concave and convex curvature of a torus induced distinguishable effects. Interestingly, cells incubated on patterned areas exhibited higher tumourigenicity. In a third example we developed a method to replicate cell-like features with a nanometre level of resolution onto polystyrene [43–45] with positive or negative aspect (Fig. 1a). We thence cultured cells on those surfaces (Fig. 1b). A small set of our results is presented in Fig. 1c [46]. Here we illustrate that cancer cells of the Ishikawa cell line, when cultured on a substrate imprinted with cell-like features, exhibited different properties to cells cultured on a flat surface. The properties included higher cytokeratin expression, higher integrin expression and, importantly, altered cell growth rate. Thus the differences in cells occurred with the same genotype and the same chemistry, *but* with a different physical architecture of the environment.

These three studies [38, 42, 46] and others, e.g. [47–49] using different models, observed that the physical characteristics of the substrate regulated central factors of cell functioning and of the cancerous phenotype, *independent* of gene modification.

## Extracellular Matrix

If the ECM provides informational signals for the cell then aspects of connectivity can be characterised between ECM proteins and cell functioning.

**Fig. 2:**
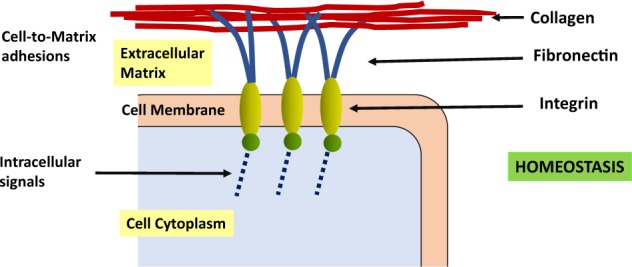
A diagram depicting the communication between polymers, collagen and fibronectin, in the ECM with the intracytoplasmic machinery of a cell via integrins

The physiological mechanism of applying forces to a cell will be largely via the ECM, which consists of a complex mixture of molecules, and has been characterised as possessing ~300 proteins [50]. The interactions between the ECM and the cell occur by various pathway complexes that involve, for example, ion channels, stretch-activated channels and cadherins [51–54]. In this review we discuss events that focus on the ECM protein polymer, collagen, and its binding with integrins at the cell membrane. A brief schema of this topic is illustrated in Fig. 2. In vivo, in the relevant environment the most important compounds in the ECM are the protein polymers, namely collagen and fibronectin [50].

Collagen is a left-handed polypeptide, three strands of which form a triple right-handed helix, called a fibril. Fibrils form bundles to construct fibres. Fibronectin is a polymer made of three types of fibronectin subunits. These are arranged to include binding sites, for example, for collagen and integrin. Fibronectin exists as an antiparallel dimer with two nearly identical monomers linked by two disulphide bonds. In the exterior of the cell, these polymers receive signals partly in the form of mechanical forces and, in response, the polymers will alter their conformations. The polymeric structures can be stretched [55] to expose cryptic binding sites, which are sometimes buried within the quaternary and tertiary structures, and thereby enhance interactive efficiency on processes that provide regulatory information targeted to a cell [56–59]. Similarly, extension of the polymer may alter coordination between multiple binding sites [60, 61]. Conversely some structural changes (e.g. elasticity, stiffness, bendability) may disrupt the binding stereochemistry and reduce the efficiency of regulation of cell behaviour.

It has been noted [62] that responses of collagen to mechanical signals can be described at four scale levels, namely molecular scale, fibrillar scale, the microscale, and the macroscale. Sequences of collagen binding partners have been mapped onto fibrillar collagen, alignment of collagen structures occurring under the influence of physical forces [62].

In fibronectin the receptors for binding to such as collagen or the sequences for binding to integrins are constructed using the arrangement of subunits. Each subunit has a molecular weight of 230–250 kDa. The type III subunit does not contain the disulphide bonds found in the other two subtypes and is therefore more readily available for unfolding under mechanical force. Fibronectin functions as fibrils, which are bundles that are 10 nm to >1 um in diameter and lengths of tens of micrometre [60]. Crosslinking of fibres is at least partly by means of hydrogen bonds. The fibronectin fibre properties derive from a mix of folding, unfolding, stretching, and contracting leading to the binding characteristics of ligands being sensitive to strain. In addition tissue properties are influenced by substantially increased rigidity of fibronectin fibre as it is extended e.g. 1–2 MPa to 50 kPa, a characteristic that influences the binding of strain-sensitive ligands. It has been observed that fibronectin can be stretched up to sevenfold. Co-assembly of collagen and fibronectin modifies proliferation and morphology by variations of spatial architecture, and mediates local variations of cell activities [63, 64].

Thus, these arrangements constitute a large repository of biological information and a centre of signalling regulation. There are both an array of receptors that are available to binding partners or not, and a plastic physical architecture of the matrix, in which forces acting on collagen and other ECM components may alter their polymeric physicochemistry, orientation and level of deposition [65, 66]. The polymers of the ECM may interact with a cell via integrins, which are cross-membrane receptors and which transduce the mechanical signal to the intracellular pathways. Thus the biophysical environment, is central to the regulation of cell behaviour. This arrangement ensures that if the environment is one that is compatible with normal healthy functioning of the cell, the cell will respond such that healthy homeostasis is maintained [19, 67]. The details that relate to such situations continue to be investigated by approaches including mathematical modelling [4].

## Mechanisms Linking Mechanics to Biology

If ECM connectivity to the cancer cell is recognised then there will be a discernible associated mechanism.

**Fig. 3:**
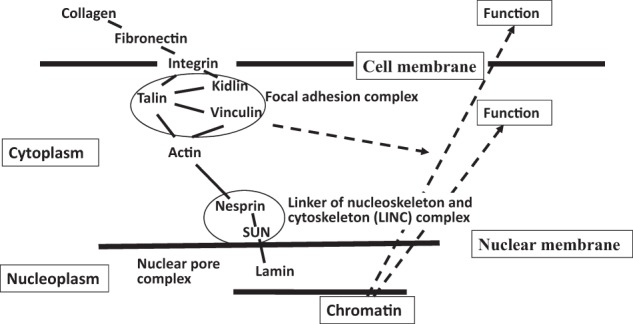
A schema of pathways available for transduction of mechanical forces in the exterior of the cell which alter the stereochemistry and biophysics of polymers in the ECM. Participation of the focal adhesion complex and the LINC complex, which connect via actin and the cytoskeleton, take the mechanical signal to the nucleus and regulatory processes that act at a gene level

A limited schema containing some pathway details by which these mechanical forces are able to alter gene expression and cell functioning is depicted in Fig. 3. As noted, the elements of the cytoskeleton, which underlies cell morphology, are components central to mechanotransduction of signals from the physical environment via the ECM [68]. Following application of mechanical forces, a series of events can be described that in coordination provide regulatory signals which alters gene expression at the nucleus [56, 65, 67, 69, 70].

Upon ECM molecule binding there is activation of integrin and the focal adhesion complex. Subsequently regulation of tensile actomyosin forces and actin reorganization occurs. The linker of nucleoskeleton and cytoskeleton (LINC) complex is conceptually the intracellular node between the cytoplasm and the nucleus. Physical propagation of forces from actin to the nucleus results in a mechanically regulated nuclear transport mechanism mediated by the LINC complex inducing chromatin organization and gene transcription [71, 72]. Some studies suggest a direct interaction between the SUN protein in the LINC complex and chromatin [73, 74]. Thereby links between the mechanical properties of the ECM and chromatin [75] are functionalised. Indeed, the processes that occur at the nucleus and modify gene activity as a result of distal mechanical forces are the subject of mathematical models [4, 65] that explore these pathways.

These signalling processes provide capability for the ECM to modulate, regulate, activate, or suppress the activity of connected genes [12]. The homeostasis of a cell is consequently dependent to a substantial extent on the properties of the ECM. The corollary of that deduction is that *disturbance of the ECM* may have potential to induce disruption of the cell’s regulation and thence its *functioning*. Thus we might predict that alteration of collagen, for example, will potentially lead to the environment being no longer compatible with healthy homeostasis, and that pathological cell behaviour results. The fact that in this circumstance the ECM and other environmental factors are unable to maintain homeostasis and do not suppress potential pathologies means that a cell expressing, say, a tumourigenic mutation, may now, in a disrupted environment, be able to proliferate, form a clone and develop into a tumour by Darwinian-type selection by fitness. Thus, whereas in the patent homeostasis the loaded oncogenes would be inhibited from expression, in the presence of a relevant disturbance of the ECM the inhibition would be removed i.e. proliferation is triggered. The environment then is a better fit for a cell containing an effective mutation and less fit for a normal cell. Thence selective survival occurs for the clone associated with cells loaded with the mutation and a tumour grows containing cells with poor proliferation control.

It was demonstrated, by examples above, that altering topography, stiffness or other physical characteristics will modify properties of the cell. Therefore, it can be suggested that disruption of collagen or other ECM elements may trigger tumourigenesis [76].

## Disrupted ECM and Cancer

If the ECM is a signalling component of homeostasis then its disruption may be associated with tumour initiation.

A variety of data on cancer associated with a range of features, including mutations, topography and orthotopic transplants, aging, and physical trauma and scar sites, can be comfortably interpreted as being consistent with the hypothesis that the physical architecture of the environment of a cell with a potentially oncogenic mutation is involved in tumour initiation.

### Mutations

If ECM is vital then mutations of ECM proteins may be associated with tumour development.

Perhaps the most easily identifiable relevant aberrations in the ECM are those caused by gene disruption. Disruption of collagen structure might be directly via *COL* mutations. Alternatively, mutations of other ECM components might occur so that interactions are altered and thence collagen tertiary or quaternary features become modified. On the other hand, some collagen mutations might be structurally benign.

(A) Notably, mutations of collagen genes have been observed to be associated with a range of cancers. For example there have been reports of mutations in *COL21A* associated with chondrosarcoma [77], of disparate mutations in glioblastoma [78], of the fusion of α chain type 1 of collagen gene and platelet-derived growth factor β gene in dermatofibrosarcoma protuberans [79], of methylation of the *COL1A2* gene and the promoter region of type IV collagen α5 and α6 chains [80], of methylation and silencing of collagen expression in hepatocellular carcinoma cell lines [80], and of methylation of the promoter-5α region of *COL1A2* in pancreatic W8 cells [80].
(B) On the other hand, it would be expected that patients with disease such as dystrophic epidermolysis bullosa (DEB) which has symptoms derived from mutations in *COL7A1* may also develop cancer. Indeed the disease is associated with increased risk of squamous cell carcinoma [81], and demands continual clinical skin examination of DEB patients. Ehrlers–Donals syndrome (EDS) also has symptoms associated with collagen mutations. However, EDS itself is rare and the uncontrolled growth that develops to a tumour requires the presence also of appropriate oncogenes (as does the initiation of all tumours) meaning that data on cancer incidence associated with EDS is sparse. Nevertheless, anecdotal evidence is available. Patients with EDS have been reported to also have malignant mesothelioma [82], gastric adenocarcinoma [83], uterine leiomyoma [84], mediastinal epithelioid haemangiomaepithelioma, and skin cancer [85, 86] as well as undefined cancer [87].

Overall these results suggest that a variation in genes associated with the ECM that lead to disruption of the biophysics of the environment, may trigger tumour initiation following the loading of classical proliferation-enhancing oncogenes.

### Orthotopic Transplant and Substrate Topography

If ECM is vital, then distinct ECM environments may be associated with a variety of tumour cell characteristics in in vivo models.

The differential effect of orthotopic transplantation site observed in several studies is consistent with the physical environment of tumour cells communicating fundamental cues to cell behaviour. An example is that an anaplastic thyroid carcinoma cell line, DRO, exhibited stronger tumourigenicity when implanted in the thyroid gland than in the subcutis. Importantly, a significant level of selectivity was demonstrated by showing there was no such difference in behaviour displayed by cells of an oral squamous cell carcinoma between transplantation to the thyroid and subcutis [88]. Also, the injection of CT-26 colon carcinoma cells into the caecal wall and spleen produced spleen and liver lesions, which exhibited responses to doxorubicin and 5-fluoracil that were distinct from those of subcutaneous tumours [89]. Similarly, small-cell lung carcinoma growing orthotopically in the lung had responses to cisplatin and mitomycin C that were distinct from subcutaneous tumours [90]. In another study cell lines of squamous cell carcinoma of the oral cavity had stronger growth patterns when orthotopically transplanted to the tongue than if they were in the ectopic subcutis [91]. Thus, differences in behaviour and drug response with transplantation at ortho- or heterotopic site indicates that genes controlling the proliferation and other cell behaviours are themselves regulated by the biophysical environment.

### Aging

**Fig. 4:**
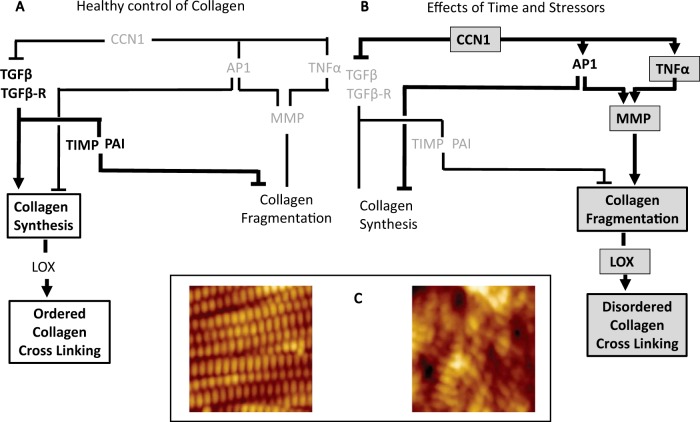
The effect of aging and other stressors on the regulation of the biophysical properties of collagen. **a** Healthy metabolic control of collagen. **b** The disrupted pathways that lead to aberrant architecture of the cell microenvironment. Grey boxes indicate factors that have increased activity in stressed tissue. **c** Atomic force microscope images of (left) young (20–30 years) skin, intact collagen fibrils are well-organized. In contrast (right) in aged skin (>80 years), collagen fibrils are fragmented and disorganized [119] (with permission of S. Karger AG)

Another way of altering collagen characteristics is by growing old. The change is clearly seen in the appearance of skin, which sags and wrinkles in older individuals as the collagen, which is tightly structured in young tissue becomes fragmented and disorganised as we age. In normal tissue TGFβ oversees a system, which regulates collagen synthesis and subsequent crosslinking of the fibrils, a process that assists in conferring the structure to the polymer that is appropriate for the tissue to maintain homeostasis. TGFβ also participates in regulating a pathway that retains the fabricated structure and inhibits the fragmentation of collagen chains by matrix metalloproteinases (MMPs) (Fig. 4a). As we age, or under the influence of other stressors, there arise increased levels of CCN1, which is a protein secreted into the matrix. CCN1 induces disruption of the homeostatic signalling of collagen synthesis and maintenance of its architectural integrity (Fig. 4b). CCN1 decreases functioning of the TGFβ-mediated processes. Further, CCN1 increases expression of AP1, a transcription factor and also increases expression of TNFα, both of which stimulate MMP activity. MMPs act on collagen polymers to induce fragmentation, and levels of lysyl oxidase (LOX) are also increased. LOX converts aldehydes on lysine residues to a highly active state and crosslinking with other converted aldehyde groups occurs. Thus the collagen fragments that result from MMP activity are connected and stabilised by LOX. Therefore, there is now a disrupted extracellular environment.

Thus, in the aged environment there has been decoupling of the homeostatic controls that are overseen by TGFβ. There is increased extent of collagen fragmentation, and the stabilisation of fragments by LOX-mediated crosslinking which are highly resistant to proteolytic cleavage. There is an increase in stiffness, and thence the homeostatic condition is destroyed, a relevant loaded oncogene is able to be expressed, and so cells can now undergo uncontrolled proliferation and resist apoptosis. Hence, the increase in stiffness and loss of healthy architecture [68, 92, 93] may not be side effects of pathological events, such as age-related cancer, rather they appear to be the *trigger* of it [57, 94, 95].

If aging is itself a factor in ECM disruption then cancer should be a disease associated with old age.

**Fig. 5:**
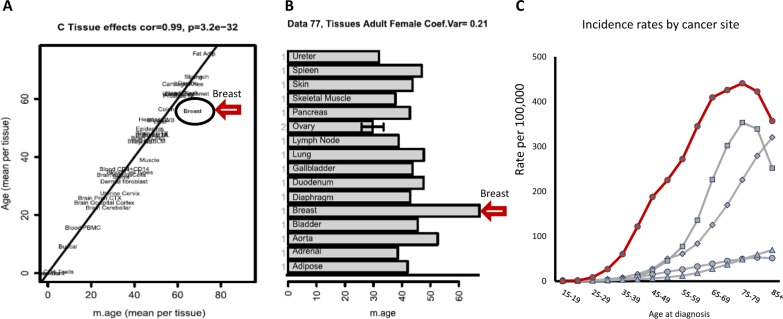
The relationship of functional (methylation) age of a tissue to the chronological age of tumour onset. **a** The discordant relationship of functional age of breast tissue (compared to other tissues) [black oval] with chronological age [96] (with permission of Springer Nature). **b** The discordant level of functional age of breast tissue (compared with other tissues) [red arrow] with chronological age in an individual woman [96] (with permission of Springer Nature). **c** Age-specific (Crude) SEER incidence rates by cancer site, all ages, all races, female, 2005–2014. The graph of onset of breast cancer [red] is earlier than that, for example, of ovary [grey square], colon and rectum [grey diamond], lung and bronchus [grey circle] and lymphocytic leukaemia [grey triangle] [14]

Establishing the details of the mechanistic relationship between aging and cell behaviour is difficult to achieve. Nevertheless, prediction of tissue functioning related to chronological age was determined in a study that investigated maintenance of gene methylation systems [96]. The results indicated that, surprisingly, breast tissue had a functioning age (methylation) older than other tissues in the body (Fig. 5a, b). If tumour genesis is associated with age-related loss of homeostasis then this observation would imply that breast cancer would appear at an earlier chronological age than do cancers of other tissues, and, in practice, this is so (Fig. 5c). In addition, in this study [96] it was observed that other biopsied tissues that contained tumours consisted of cells which had aged faster than healthy tissue in the same individuals.

Therefore, it may be postulated that in young healthy tissue the environment is such that expression of oncogenic mutations is suppressed and clones of normal cells develop—the environment favours normal cells, healthy cells outcompete those with cancerous mutations, and homeostasis is maintained. On the other hand, when the environment is disrupted by aging or other factors, there is aberrant compression, stiffness, extracellular tension, elasticity and topography of the tissue. Thence the alteration of the ECM physical structures and stereochemistry induce either directly or indirectly modification of ECM activities and cell behaviour. For example (i) *Smoking*: Smoking affects ECM regulation of collagen by altering factors such as expression of MMP, and TIMP [97]. In addition, there are physical effects whereby the micro- and nano-scale particulate matter may interact act with dynamic mechanical forces on polymers, such as collagen [98]. Further, particles may clog the filtering function of a structured ECM thereby altering ECM-mediated controls, both mechanical and biochemical, thus inducing remodelling of the ECM [99]. (ii) *Radiation*: Radiation may reduce ECM stiffness partly by eliciting cleavage of peptide bonds in the backbone of the collagen polymer. It has been proposed that collagen in aged individuals will have accumulated damage, including single bond cleavage events, thereby causing changes in the collagen primary structure resulting in destabilisation of the collagen architecture [100, 101]. (iii) *Inflammation*: The persistence of inflammation at the injury site sustains ECM disorganization, including that of collagen, partly by activating MMPs. Degradation of the ECM and alteration of collagen deposition activates inflammation-related pathways [102]. Concurrently with matrix degradation, there is inflammation-mediated downregulation of growth and transcription factors that mediate ECM synthesis, such as TGF-$\beta$. These changes alter the mechanical properties of the tissue and thereby alter cell behaviour [103]. The effect is delayed healing and repair to tissue topography [102] and provision of a tumour-favourable niche [104]. (iv) *Bacteria*: Several pathways in natural wound healing are similar to those of tumour development. Wound microbial colonisation may lead to inflammatory states, and an optimal environment for malignant cells to develop as tumours may develop, which are associated with altered gene expression for several ECM proteins and receptors including collagen [105].

As a result of ECM disruption and remodelling there are alterations in receptor conformations and activity of signalling pathways [66]; now the inhibition of mutated oncogenic gene expression is destroyed, and tumours develop. Thereby fitness of a cell to its environment is a dynamic property imposed by the environment, not by mutations directing an inherent cell characteristic [19, 20].

### Physical Trauma and Scars

If biological ECM-related factors (such as genes of protein polymers and physiological environments) affect tumour initiation, then physical alteration of the ECM might also be associated with tumour initiation.

Following the recognition that many observations are consistent with the biomechanical environment being of prime importance in tumour genesis we are led to question whether physical factors, such as wounds and scars where tissue organisation has been disrupted, may provide an anatomical focus for enhanced tumour growth. Although the topic was considered in some early anecdotal reports there have been few robust studies, possibly due to the investigative emphasis in cancer development being on gene-associated mechanisms. An early investigation of cancer at a prior site of benign lesion [106] reviewed reports related to non-malignant lesions in skin, in mucous membrane of gastrointestinal tract and genitourinary tract, and in epidermal components of teratomas. The cancer types were squamous cell and transitional cell, and only a few of the cancers in this study were adenocarcinoma and melanoma. It was concluded that physical factors including distortion, tensile stress, and dilation or a group of disturbances such as inflammation, scarring and tensile stress provided an environment compatible with cancer initiation, and not only was destruction of the epithelium involved. Although the latent period was usually in the range 10–70 years some cancers developed rapidly after weeks or months, most often in already atrophic or keratotic aged skin. The report suggested that orderly arrangement (operative scars, colostomy openings, and corns and calluses) was much less often associated with eventual cancer [106]. In a study conducted on women between 1961 and 1975 it was reported that twelve women presented with breast cancer at the site of old surgical scars [107]. The scars were vascularized, poorly organized and the areas were more liable to ulceration, degeneration and malignant transformation. The authors were persuaded that the women presented with a distinct entity, namely ‘*scar-related carcinomas*’. Another investigation, of trauma on development of basal cell carcinoma included burns, sharp trauma, blunt trauma, chicken pox scars and vaccination sites [108]. Between 1979 and 1986 the authors evaluated 1774 basal cell carcinoma lesions that were treated by Mohs surgery and noted that 129 (7.3%) had a previous history of trauma. Patients with trauma-related tumours were slightly younger, the lesions were larger pre- and post-operation, and were deeper (*p* < 0.001, <0.05). The results therefore suggested a difference in behaviour between tumours on areas with previous trauma from those without. A fourth study also considered a connection between physical trauma and breast cancer [109]. The authors recruited 134 control women without cancer and 67 women with cancer. Sixteen (12%) of the control women reported experiencing a trauma on her breast in the previous 5 years and 35 (52%) of the case women (*p* < 0.001). This wide statistical difference convinced the authors that the trauma was a *cause* of the cancer. Further, two reports involving hepatocellular function also suggested injury may be an important factor in tumour growth. In the first, a review [110], the components of ‘the wound healing, chronic fibrosis, and cancer progression triad’ are noted, in which injury is caused by such as alcohol, hepatitis viruses, excess fat or cholestasis, and an inflammatory response ensues. Then, consequently recruited hepatic stellate cells convert to myofibroblasts, which produce copious amounts of ECM including fibrillary collagen, fibrogenesis takes place and, as a consequence, malignant transformation occurs. The second, a study [111] focused on a single aetiology, chronic HBV infection. The severity of fibrosis and inflammation specimens were scored using surgical resection in the non-neoplastic liver and it was observed that the patients with Ishak stage 6 had poor overall and recurrence-free survival when compared with patients with Ishak stage 1–5 (*p* < 0.01). This overall set of results provides further supporting evidence that the biophysical characteristics of the environment affects tumour *genesis*.

**Fig. 6:**
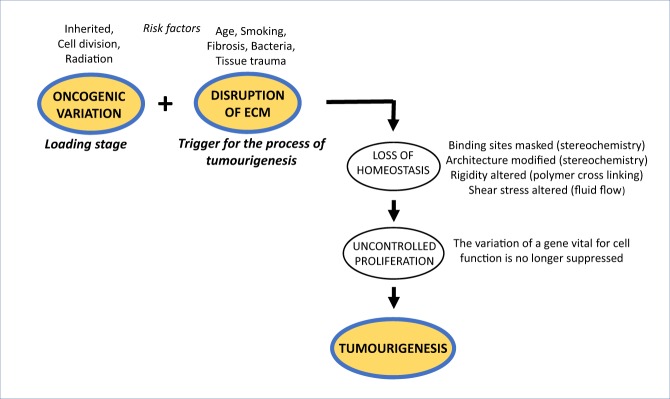
The overall scheme of the load-trigger hypothesis for tumour initiation. An oncogene is loaded, and triggering of the event of tumourigenesis occurs when disruption of the ECM results in loss of homeostasis

The observations that report increased tumour development at sites where the ECM has been physically altered provide statistical evidence that goes beyond anecdotal reports. The observations are consistent with disturbance being a fundamental factor [93] in tumour initiation. Further, an association between keloids and cancer has been reported [112, 113]. There are, of course, many occasions where scars are not sites of subsequent tumour growth, probably reflecting that for cancerous behaviour to occur, the disturbance of ECM must be *accompanied* by a loaded tissue-specific oncogene that has potential to elicit uncontrolled proliferation in *that* tissue (Fig. 6).

## Reversion to a Healthy Phenotype

If aberrant biophysics is central to tumour initiation, then repairing the altered biophysics or its signalling pathway might induce tumour reversion.

If physical and mechanical properties of the cellular environment are key in controlling cell behaviour, then the corollary is that alteration of the environment to one that is compatible with healthy homeostasis might redirect a tumourigenic cell to a normal phenotype. The idea of seeking clinical targets in ECM dysfunction has attracted a significant congregation (e.g. [114–116]). The concept of targeting the extracellular environment for clinical manipulation and providing an alternative approach rather than neutralising mutations of a cell pathway gene, has been noted [117]. In this regard speculative suggestions have been made on the possibility of pharmacologically altering activity in the ECM of, for example, LOX [118], CCN1 [119], fibronectin [64] or FAK [120], which may provide accessible targets. Intriguingly, it has been suggested [101] that their studies indicated that therapeutic radiation may be used to beneficially modify ECM mechanical properties. Further, mechanical signals, including tension, compression, and paper taping may be effective in targeting the development of a fibrotic ECM [121]. Such therapies may enable cancer progression to be halted and be modified to being a manageable chronic but non-lethal disease [122]. If the physical landscape itself is to be a target for treatment then the possibility of tissue engineering being effective is a topic for exciting speculation [123].

The concept of biophysically-induced reversion of malignant phenotype to normal phenotype has been investigated in a variety of contexts with evidence of success. If aberrant *communication* between the biophysical environment and the cell is at the foundation of a cell exhibiting a malignant phenotype, then interrupting that communication may be expected to block the transmission of pathological instructions [124]. During investigation of cells derived from a benign breast lesion it was noticed that a clone exhibiting a malignant phenotype had arisen, in which, instead of organised formation of acinar structures typical of healthy breast tissue, the cells were disorganised and underwent uncontrolled proliferation. A mutation in an integrin gene was identified and antibodies against the mutated integrin gene product were produced. When the cells were incubated with the antibody the cells reverted to a healthy phenotype and formed ordered acinar structures, reverting to the phenotype of the cells from which the clone was originally derived [124]. Further investigation of this model revealed that cytoskeletal rearrangement was mediated by pathways including EGFR, MEK, Akt, FAK, and RhoA/ROCK signalling, which are important to constructing the phenotype and acinar formation [125]. Another study [126] compared cells of a breast cell line (MDA-MB-435S) that were incubated in environments of distinct compositions. When cells were incubated in Matrigel, a common substrate for cell culture experiments, the cell arrangements were disordered cell clusters with polymorphic nuclei and diffuse beta-catenin expression. On the other hand, cells incubated with the self-assembling peptide RADA16 exhibited a healthy phenotype of acinar structures, uniform, polarised nuclei and β-catenin localised at cell-to-cell junctions. The authors concluded that the selection of malignant or non-malignant phenotype when the cells were incubated with one or the other condition was dependent on the biophysical environment rather than alteration of immutable genes. In a further study [80] using the pancreatic cell line, W8, the characteristics of the pathological phenotype, which results from a mutation of a collagen gene, were ameliorated by successful transfection of a functioning gene. Following transfection, cells adhered more firmly to the substratum, maintained slower growth kinetics, and also exhibited lower tumourigenicity in nude mice. Thus repairing the aberration of collagen production inhibited transformation to the malignant phenotype.

These observations together indicate that the possibility of turning cancer into healthy tissue has been plausibly and strongly illustrated. However, the considerable absence of details still to be acquired in this field is illustrated by the behaviour of cancer cells introduced into orthoptic tissues, where the biophysical environment clearly affects behaviour but does not routinely induce unequivocal reversion. However, some new research supports the concept of executive control of cell function residing in the environment, which can be recruited to revert aberrant cell behaviour to that of a healthy cell. Studies with rat brain oligodendrocyte progenitor cells demonstrated that cells with age-related dysfunction reverted to young healthy behaviour when cultured in a suitable environment of the low stiffness that is associated with that in young rats [127].

## Discussion

We restricted this paper to discussion of early stage cancer, and excluded changes associated with progression and spread to a metastatic state. Thus, factors involving growth of established tumours, passage of cells through vessel membranes, or invasion of cancers into diverse tissues, were set aside as possibly having underlying mechanisms different from those of tumour initiation. The review provides a template for an alternative approach to understanding tumourigenesis (Fig. 6). The process begins by the loading of an oncogene, either inherited or somatic. Under controlled homeostasis the gene is suppressed. The ECM regulates cell activity via intracellular pathways that are well described by research of healthy and non-cancer pathologies. Later, homeostasis may be upset by factors such as age, smoking, physical injury and so on. The variation in controlling elements triggers the expression of the oncogene (Fig. 6). These considerations are becoming more vital in the face of recent observations that in healthy cells there is considerable and widespread presence of particular mutations that have hitherto been associated with cancerous phenotype [128, 129].

This review provided a distinct context for discussing tumour initiation and a triggering mechanism. Studies aimed to directly confirm (or refute) the notion are required. Nevertheless, we here provide a large suite of data and observations that are consistent with the suggestion that the primary trigger for malignancy is biological physics in the presence of a tumourigenic gene variation. The review noted, for example, that biological disruption of the ECM is temporally associated with cancer development [14], the time profiles of mutations or cell divisions do not parallel that of cancer incidence [19], there is an association between ‘aged’ tissue and tumourigenesis in individuals [96], and reversal is possible by targeted interference with the ECM [124].

Importantly, there are clinical examples where details are recently available enabling a demonstration of how this approach can explain a variety of observations. The first example is a study [130] on high-grade serous ovarian carcinoma, the most common (75%) ovarian cancer. The study determined that p53 signatures and serous tubal intraepithelial carcinomas are precursors of ovarian carcinoma; they were detectable in fallopian tubes but full malignant behaviour of oncogenic variations (including of BRCA) was not evident until the cells had migrated to another environment in the ovary and surrounding tissues. The second environment supports survival and expansion, and is the site of inflammation and fibrosis [131]. Thus in one environment an oncogenic mutation is not expressed but in another, fibrotic, environment it is and cancer develops i.e. the malignant process is first loaded and then triggered. The second example is related to asbestos-associated malignant mesothelioma. The latency from first exposure to asbestos is long [132, 133] with a median period of about 50 years [134, 135]. Inherited predisposition associated with asbestos-related malignant mesothelioma [133, 136] has been detected and germline mutations identified [137] in a number of individuals with onset still after a long latency period. There is an inflammatory response, with alteration of collagen architecture and deposition in the local environment [136, 138–140] and deep stromal invasion [132]. Notably the changes are accompanied by increased methylation [132], which points to functional aging [96]. Again, loading followed by triggering is apparent. A third example is that of the two-stage initiation of childhood acute lymphoblastic leukaemia [141]. An oncogenic mutation is acquired during fetal development in utero, but the development of a bacteria-induced change [141] in the extracellular environment [105, 142, 143] is necessary for actually triggering cancerous behaviour. Thus, the careful dissection of the pathology produced observations that are, again, consistent with the process suggested in this review. Together these observations indicate characteristics reported in diverse clinical studies that can be explained by a load-trigger mechanism.

The variation in expression of a particular oncogene between tissues may be at least partly controlled at the level of transcription factors, which are under environment-dependent control, a characteristic that confers tissue specificity on regulation systems [144]. Transcription factors have been observed to respond to mechanical signals [145], and their further binding to enhancer DNA elements [146] can provide exquisite sensitivity to the expression of the functioning gene [147]. Thus, biophysical variation affecting these factors may alter tumourigenic risk at a particular site [144, 145]. The tissue specificity of mechanosensitive signalling elements may therefore also explain, for example, the apparently low incidence of trauma-induced cancer. Further, as a result of tissue-specific modulation, the potency of the mechanism discussed in this paper may vary across tumour sites and other initiation processes may possibly be active.

Thus, the review adduces evidence of connections between the scales, from molecular scale with collagen as exemplar to nanotopography and all the way up to macroscale at tissue level. Some of the links have been only sparsely previously remarked upon, and often were only indirectly considered. It may be a symptom of both the certainty that mechanotransduction is important to cell behaviour and the hesitancy of articulating defined roles to the physical environment that mathematic modellers are attracted to the topic. Theoretical models are able to postulate the effects of the forces towards an understanding at the molecular or atomic level. It is plausible to recognise events so that stochastic and dynamic spatial states that are physiologically accurate are described [4].

The initiation of the tumour may be established by positive feedback by, for example, increased tension as a result of ECM signals affecting the cytoskeleton, which itself alters the connection between cell and substrate [5, 65]. To analyse pathway and time dynamics that are involved, the intersection between signalling and mechanics is being computationally modelled by several approaches [148]. Therefore the ECM appears to be a potential route for anticancer treatments. If properly selected, the targeted alteration will inhibit pathological activity. It may include repair of gene function (e.g. of a collagen gene variant), altering ECM composition (e.g. by affecting collagen production, via TGF), modifying polymer or protein chemistry or stereochemistry (e.g. reducing crosslinking and modulating rigidity or manipulating exposure of cryptic sites), or affecting collagen architecture and environment topography (e.g. by variation in LOX or MMP presence). The strategic targeting of upstream steps (at the ECM and its interactions with cells) may have substantially reduced risk of associated morbidities than with a drug that inhibits general proliferation, especially at the very early stages which is the focus of this review. The strength of the already assembled evidence from a range of studies with assorted aims encourages such a focus in new studies. Such studies that obtained details of ECM functioning would be highly valued because additional direct evidence is still required, such as for collagen density, alignment and networked architecture [66, 149]. The concept that a permissive environment is fundamental to tumour initiation has been nebulously proposed for several years. This review provides a coherent skeleton for discussing this opinion in the context of contemporary knowledge of biochemistry, genetics and biophysics.

## References

[1] Pickup MW, Mouw JK, Weaver VM (2014). The extracellular matrix modulates the hallmarks of cancer. EMBO Reports.

[2] Plodinec M, Loparic M, Monnier CA, Obermann EC, Zanetti-Dallenbach R, Oertle P, Hyotyla JT, Aebi U, Bentires-Alj M, Lim RY, Schoenenberger CA (2012). The nanomechanical signature of breast cancer. Nature Nanotechnology.

[3] Walker C, Mojares E, Del Rio Hernandez A (2018). Role of extracellular matrix in development and cancer progression. International Journal of Molecular Sciences.

[4] Mak M, Kim T, Zaman MH, Kamm RD (2015). Multiscale mechanobiology: computational models for integrating molecules to multicellular systems. Integrative Biology.

[5] Emon B, Bauer J, Jain Y, Jung B, Saif T (2018). Biophysics of tumor microenvironment and cancer metastasis—a mini review. Computational and Structructural Biotechnology Journal.

[6] Lopez-Lazaro M (2018). The stem cell division theory of cancer. Critical Reviews in Oncology Hematology.

[7] Takeshima H, Ushijima T (2019). Accumulation of genetic and epigenetic alterations in normal cells and cancer risk. NPJ Precision Oncology.

[8] Mueller MM, Fusenig NE (2004). Friends or foes—bipolar effects of the tumour stroma in cancer. Nature Reviews Cancer.

[9] Khan Z, Marshall JF (2016). The role of integrins in TGF beta activation in the tumour stroma. Cell and Tissue Research.

[10] Artandi SE, Attardi LD (2005). Pathways connecting telomeres and p53 in senescence, apoptosis, and cancer. Biochemical and Biophysical Research Communications.

[11] Tashiro H, Brenner MK (2017). Immunotherapy against cancer-related viruses. Cell Research.

[12] Bissell MJ, Hines WC (2011). Why don’t we get more cancer? A proposed role of the microenvironment in restraining cancer progression. Nature Medicine.

[13] National Cancer Institute (2017a). BRCA1 and BRCA2: cancer risk. National Cancer Institute. http://www.cancer.gov/about-cancer/causes-prevention/genetics/brca-fact-sheet#q2.

[14] National Cancer Institute (2017b). Cancer incidence. National Cancer Institute. http://seer.cancer.gov/faststats/selections.php?#Output.

[15] Drost JB, Lee WR (1995). Biological basis of germline mutation: comparisons of spontaneous germline mutation rates among drosophila, mouse, and human. Environmental and Molecular Mutagenesis.

[16] Simpson AJ (1997). The natural somatic mutation frequency and human carcinogenesis. Advances in Cancer Research.

[17] Aunan JR, Cho WC, Soreide K (2017). The biology of aging and cancer: a brief overview of shared and divergent molecular hallmarks. Aging and Disease.

[18] Lopez-Lazaro M (2018). Cancer etiology: variation in cancer risk among tissues is poorly explained by the number of gene mutations. Genes Chromosomes and Cancer.

[19] Rozhok AI, DeGregori J (2015). Toward an evolutionary model of cancer: Considering the mechanisms that govern the fate of somatic mutations. Proceedings of the National Academy of Sciences of the United States of America.

[20] Liggett LA, DeGregori J (2017). Changing mutational and adaptive landscapes and the genesis of cancer. Biochimica et Biophysica Acta Reviews on Cancer.

[21] McAllister P (2010). Manthropology: the science of why the modern male is not the man he used to be..

[22] Tipton CM (2014). The history of “Exercise Is Medicine” in ancient civilizations. Advances in Physiology Education.

[23] Becker JL, Souza GR (2013). Using space-based investigations to inform cancer research on Earth. Nature Reviews Cancer.

[24] Lewis ML, Cubano LA, Zhao B, Dinh HK, Pabalan JG, Piepmeier EH, Bowman PD (2001). cDNA microarray reveals altered cytoskeletal gene expression in space-flown leukemic T lymphocytes (Jurkat). FASEB Journal.

[25] Guo F, Li Y, Liu Y, Huang J, Zhang Z, Wang J, Li Y, Hu J, Li G (2012). Identification of genes associated with tumor development in CaSki cells in the cosmic space. Molecular Biology Reports.

[26] Kafri R, Levy J, Ginzberg MB, Oh S, Lahav G, Kirschner MW (2013). Dynamics extracted from fixed cells reveal feedback linking cell growth to cell cycle. Nature.

[27] Tzur A, Kafri R, LeBleu VS, Lahav G, Kirschner MW (2009). Cell growth and size homeostasis in proliferating animal cells. Science.

[28] Chen CS, Mrksich M, Huang S, Whitesides GM, Ingber DE (1997). Geometric control of cell life and death. Science.

[29] Clark AG, Paluch E (2011). Mechanics and regulation of cell shape during the cell cycle. Results and Problems in Cell Differentiation.

[30] Vandenburgh HH (1992). Mechanical forces and their second messengers in stimulating cell growth in vitro. American Journal of Physiology.

[31] van Oers RF, Ruimerman R, Tanck E, Hilbers PA, Huiskes R (2008). A unified theory for osteonal and hemi-osteonal remodeling. Bone.

[32] Sridharan A, Rajan SD, Muthuswamy J (2013). Long-term changes in the material properties of brain tissue at the implant-tissue interface. Journal of Neural Engineering.

[33] Leal-Egana A, Fritsch A, Heidebrecht F, Diaz-Cuenca A, Nowicki M, Bader A, Kas J (2012). Tuning liver stiffness against tumours: an in vitro study using entrapped cells in tumour-like microcapsules. Journal of the Mechanical Behavior of Biomedical Materials.

[34] Choi JK, Agarwal P, Huang H, Zhao S, He X (2014). The crucial role of mechanical heterogeneity in regulating follicle development and ovulation with engineered ovarian microtissue. Biomaterials.

[35] Chromiak JA, Vandenburgh HH (1994). Mechanical stimulation of skeletal muscle cells mitigates glucocorticoid-induced decreases in prostaglandin production and prostaglandin synthase activity. Journal of Cellular Physiology.

[36] Delvoye P, Wiliquet P, Leveque JL, Nusgens BV, Lapiere CM (1991). Measurement of mechanical forces generated by skin fibroblasts embedded in a three-dimensional collagen gel. Journal of Investigative Dermatology.

[37] Paz H, Pathak N, Yang J (2014). Invading one step at a time: the role of invadopodia in tumor metastasis. Oncogene.

[38] Lee J, Abdeen AA, Wycislo KL, Fan TM, Kilian KA (2016). Interfacial geometry dictates cancer cell tumorigenicity. Nature Materials.

[39] Northey JJ, Przybyla L, Weaver VM (2017). Tissue force programs cell fate and tumor aggression. Cancer Discovery.

[40] Lemanne D, Cassileth B, Gubili J (2013). The role of physical activity in cancer prevention, treatment, recovery, and survivorship. Oncology.

[41] Ashcraft KA, Peace RM, Betof AS, Dewhirst MW, Jones LW (2016). Efficacy and mechanisms of aerobic exercise on cancer initiation, progression, and metastasis: a critical systematic review of in vivo preclinical data. Cancer Research.

[42] Guo CL, Ouyang M, Yu JY, Maslov J, Price A, Shen CY (2012). Long-range mechanical force enables self-assembly of epithelial tubular patterns. Proceedings of the National Academy of Sciences of the United States of America.

[43] Murray LM, Nock V, Evans JJ, Alkaisi MM (2016). The use of substrate materials and topography to modify growth patterns and rates of differentiation of muscle cells. Journal of Biomedical Materials Research Part A.

[44] Mutreja I, Woodfield TB, Sperling S, Nock V, Evans JJ, Alkaisi MM (2015). Positive and negative bioimprinted polymeric substrates: new platforms for cell culture. Biofabrication.

[45] Muys JJ, Alkaisi MM, Evans JJ (2006). Cellular replication and atomic force microscope imaging using a UV-Bioimprint technique. Nanomedicine.

[46] Tan LH, Sykes PH, Alkaisi MM, Evans JJ (2015). The characteristics of Ishikawa endometrial cancer cells are modified by substrate topography with cell-like features and the polymer surface. International Journal of Nanomedicine.

[47] Sariisik E, Docheva D, Padula D, Popov C, Opfer J, Schieker M, Clausen-Schaumann H, Benoit M (2013). Probing the interaction forces of prostate cancer cells with collagen I and bone marrow derived stem cells on the single cell level. PLoS ONE.

[48] Tang, X., Kuhlenschmidt, TB., Zhou, J., Bell, P., Wang, F., Kuhlenschmidt, MS., & Saif, TA. (2010) Mechanical force affects expression of an in vitro metastasis-like phenotype in HCT-8 cells. *Biophys J*. *99*, 2460–9. 10.1016/j.bpj.2010.08.034 PMC2955412 20959086

[49] McKenzie AJ, Hicks SR, Svec KV, Naughton H, Edmunds ZL, Howe AK (2018). The mechanical microenvironment regulates ovarian cancer cell morphology, migration, and spheroid disaggregation. Scientific Reports.

[50] Hynes RO, Naba A (2012). Overview of the matrisome–an inventory of extracellular matrix constituents and functions. Cold Spring Harbor Perspectives in Biology.

[51] Poole K, Moroni M, Lewin GR (2015). Sensory mechanotransduction at membrane-matrix interfaces. Pflugers Archiv.

[52] Jiao R, Cui D, Wang SC, Li D, Wang YF (2017). Interactions of the mechanosensitive channels with extracellular matrix, integrins, and cytoskeletal network in osmosensation. Frontiers in Molecular Neuroscience.

[53] Brucher BL, Jamall IS (2014). Cell-cell communication in the tumor microenvironment, carcinogenesis, and anticancer treatment. Cellular Physiology and Biochemistry.

[54] Litan A, Langhans SA (2015). Cancer as a channelopathy: ion channels and pumps in tumor development and progression. Frontiers in Cellular Neuroscience.

[55] Graham JS, Vomund AN, Phillips CL, Grandbois M (2004). Structural changes in human type I collagen fibrils investigated by force spectroscopy. Experimental Cell Research.

[56] Roca-Cusachs P, Iskratsch T, Sheetz MP (2012). Finding the weakest link: exploring integrin-mediated mechanical molecular pathways. Journal of Cell Science.

[57] Lopez JI, Mouw JK, Weaver VM (2008). Biomechanical regulation of cell orientation and fate. Oncogene.

[58] Orgel JP, Antipova O, Sagi I, Bitler A, Qiu D, Wang R, Xu Y, San Antonio JD (2011). Collagen fibril surface displays a constellation of sites capable of promoting fibril assembly, stability, and hemostasis. Connective Tissue Research.

[59] Perumal S, Antipova O, Orgel JP (2008). Collagen fibril architecture, domain organization, and triple-helical conformation govern its proteolysis. Proceedings of the National Academy of Sciences of the United States of America.

[60] Bradshaw MJ, Smith ML (2014). Multiscale relationships between fibronectin structure and functional properties. Acta Biomaterialia.

[61] Hoop CL, Zhu J, Nunes AM, Case DA, Baum J (2017). Revealing accessibility of cryptic protein binding sites within the functional collagen fibril. Biomolecules.

[62] Sherman VR, Yang W, Meyers MA (2015). The materials science of collagen. Journal of the Mechanical Behavior of Biomedical Materials.

[63] Sevilla CA, Dalecki D, Hocking DC (2013). Regional fibronectin and collagen fibril co-assembly directs cell proliferation and microtissue morphology. PLoS ONE.

[64] Wang K, Seo BR, Fischbach C, Gourdon D (2016). Fibronectin mechanobiology regulates tumorigenesis. Cellular and Molecular Bioengineering.

[65] Northcott JM, Dean IS, Mouw JK, Weaver VM (2018). Feeling stress: the mechanics of cancer progression and aggression. Frontiers in Cell and Developmental Biology.

[66] Malandrino A, Trepat X, Kamm RD, Mak M (2019). Dynamic filopodial forces induce accumulation, damage, and plastic remodeling of 3D extracellular matrices. PLOS Computational Biology.

[67] Humphrey JD, Dufresne ER, Schwartz MA (2014). Mechanotransduction and extracellular matrix homeostasis. Nature Reviews Molecular Cell Biology.

[68] Nagelkerke A, Bussink J, Rowan AE, Span PN (2015). The mechanical microenvironment in cancer: how physics affects tumours. Seminars in Cancer Biology.

[69] Jahed Z, Shams H, Mehrbod M, Mofrad MR (2014). Mechanotransduction pathways linking the extracellular matrix to the nucleus. International Review of Cell and Molecular Biology.

[70] Bouvard D, Pouwels J, De Franceschi N, Ivaska J (2013). Integrin inactivators: balancing cellular functions in vitro and in vivo. Nature Reviews Molecular Cell Biology.

[71] Dechat T, Adam SA, Taimen P, Shimi T, Goldman RD (2010). Nuclear lamins. Cold Spring Harbor Perspectives in Biology.

[72] Shivashankar GV (2011). Mechanosignaling to the cell nucleus and gene regulation. Annual Review of Biophysics.

[73] Horn HF, Brownstein Z, Lenz DR, Shivatzki S, Dror AA, Dagan-Rosenfeld O, Friedman LM, Roux KJ, Kozlov S, Jeang KT, Frydman M, Burke B, Stewart CL, Avraham KB (2013). The LINC complex is essential for hearing. Journal of Clinical Investigation.

[74] Yoshida T, Landhuis E, Dose M, Hazan I, Zhang J, Naito T, Jackson AF, Wu J, Perotti EA, Kaufmann C, Gounari F, Morgan BA, Georgopoulos K (2013). Transcriptional regulation of the Ikzf1 locus. Blood.

[75] Iyer KV, Pulford S, Mogilner A, Shivashankar GV (2012). Mechanical activation of cells induces chromatin remodeling preceding MKL nuclear transport. Biophysical Journal.

[76] Zou X, Feng B, Dong T, Yan G, Tan B, Shen H, Huang A, Zhang X, Zhang M, Yang P, Zheng M, Zhang Y (2013). Up-regulation of type I collagen during tumorigenesis of colorectal cancer revealed by quantitative proteomic analysis. Journal of Proteomics.

[77] Ikeda K, Iyama K, Ishikawa N, Egami H, Nakao M, Sado Y, Ninomiya Y, Baba H (2006). Loss of expression of type IV collagen alpha5 and alpha6 chains in colorectal cancer associated with the hypermethylation of their promoter region. The American Journal of Pathology.

[78] Merid SK, Goranskaya D, Alexeyenko A (2014). Distinguishing between driver and passenger mutations in individual cancer genomes by network enrichment analysis. BMC Bioinformatics.

[79] Llombart B, Serra-Guillen C, Monteagudo C, Lopez Guerrero JA, Sanmartin O (2013). Dermatofibrosarcoma protuberans: a comprehensive review and update on diagnosis and management. Seminars in Diagnostic Pathology.

[80] Sengupta PK, Smith EM, Kim K, Murnane MJ, Smith BD (2003). DNA hypermethylation near the transcription start site of collagenalpha2(I) gene occurs in both cancer cell lines and primary colorectal cancers. Cancer Research.

[81] Fine JD, Johnson LB, Weiner M, Li KP, Suchindran C (2009). Epidermolysis bullosa and the risk of life-threatening cancers: the National EB Registry experience, 1986–2006. Journal of the American Academy of Dermatology.

[82] Bisconti M, Bisetti A, Bidoli P (2000). Malignant mesothelioma in subjects with Marfan's syndrome and Ehlers-Danlos syndrome: only an apparent association?. Respiration.

[83] Kanechorn Na Ayuthaya R, Patthamapasphong N, Sura T, Niumpradit N, Trachoo O (2008). Ehlers-Danlos syndrome type IV with gastric adenocarcinoma. Journal of the Medical Association of Thailand.

[84] Wegrowski Y, Bellon G, Quereux C, Maquart FX (1999). Biochemical alterations of uterine leiomyoma extracellular matrix in type IV Ehlers-Danlos syndrome. American Journal of Obstetrics and Gynecology.

[85] Lichtenstein JR (1975). Skin cancer in a patient with the Ehlers-Danlos syndrome. Birth Defects Original Article Series.

[86] Zhao B, Pritchard JR (2016). Inherited disease genetics improves the identification of cancer-associated genes. PLOS Genetics.

[87] Oderich GS, Panneton JM, Bower TC, Lindor NM, Cherry KJ, Noel AA, Kalra M, Sullivan T, Gloviczki P (2005). The spectrum, management and clinical outcome of Ehlers-Danlos syndrome type IV: a 30-year experience. Journal of Vascular Surgery.

[88] Kim S, Park YW, Schiff BA, Doan DD, Yazici Y, Jasser SA, Younes M, Mandal M, Bekele BN, Myers JN (2005). An orthotopic model of anaplastic thyroid carcinoma in athymic nude mice. Clinical Cancer Research.

[89] Wilmanns C, Fan D, O'Brian CA, Bucana CD, Fidler IJ (1992). Orthotopic and ectopic organ environments differentially influence the sensitivity of murine colon carcinoma cells to doxorubicin and 5-fluorouracil. International Journal of Cancer.

[90] Kuo TH, Kubota T, Watanabe M, Furukawa T, Kase S, Tanino H, Saikawa Y, Ishibiki K, Kitajima M, Hoffman RM (1993). Site-specific chemosensitivity of human small-cell lung carcinoma growing orthotopically compared to subcutaneously in SCID mice: the importance of orthotopic models to obtain relevant drug evaluation data. Anticancer Research.

[91] Myers JN, Holsinger FC, Jasser SA, Bekele BN, Fidler IJ (2002). An orthotopic nude mouse model of oral tongue squamous cell carcinoma. Clinical Cancer Research.

[92] Kai F, Laklai H, Weaver VM (2016). Force matters: biomechanical regulation of cell invasion and migration in disease. Trends in Cell Biology.

[93] Feller L, Khammissa RAG, Lemmer J (2017). Biomechanical cell regulatory networks as complex adaptive systems in relation to cancer. Cancer Cell International.

[94] Levental KR, Yu H, Kass L, Lakins JN, Egeblad M, Erler JT, Fong SF, Csiszar K, Giaccia A, Weninger W, Yamauchi M, Gasser DL, Weaver VM (2009). Matrix crosslinking forces tumor progression by enhancing integrin signaling. Cell.

[95] Doyle AD, Yamada KM (2016). Mechanosensing via cell-matrix adhesions in 3D microenvironments. Experimental Cell Research.

[96] Horvath S (2013). DNA methylation age of human tissues and cell types. Genome Biology.

[97] Knuutinen A, Kokkonen N, Risteli J, Vahakangas K, Kallioinen M, Salo T, Sorsa T, Oikarinen A (2002). Smoking affects collagen synthesis and extracellular matrix turnover in human skin. British Journal of Dermatology.

[98] Masilamani V, AlZahrani K, Devanesan S, AlQahtani H, AlSalhi MS (2016). Smoking induced hemolysis: spectral and microscopic investigations. Scientific Reports.

[99] Engin AB, Nikitovic D, Neagu M, Henrich-Noack P, Docea AO, Shtilman MI, Golokhvast K, Tsatsakis AM (2017). Mechanistic understanding of nanoparticles' interactions with extracellular matrix: the cell and immune system. Particle and Fibre Toxicology.

[100] Jariashvili K, Madhan B, Brodsky B, Kuchava A, Namicheishvili L, Metreveli N (2012). UV damage of collagen: insights from model collagen peptides. Biopolymers.

[101] Miller J, Borde B, Bordeleau F, Zanotelli M, LaValley D, Parker D, Bonassar L, Pannullo S, Reinhart-King C (2018). Clinical doses of radiation reduce collagen matrix stiffness. APL Bioengineering.

[102] Thankam FG, Roesch ZK, Dilisio MF, Radwan MM, Kovilam A, Gross RM, Agrawal DK (2018). Association of inflammatory responses and ECM disorganization with HMGB1 upregulation and NLRP3 inflammasome activation in the injured rotator cuff tendon. Scientific Reports.

[103] Maldonado M, Nam J (2013). The role of changes in extracellular matrix of cartilage in the presence of inflammation on the pathology of osteoarthritis. BioMed Research International.

[104] Druso JE, Fischbach C (2018). Biophysical properties of extracellular matrix: linking obesity and cancer. Trends in Cancer.

[105] Park SS, Izadjoo MJ (2014). Wound infections and healing: are they contributing factors for carcinogenesis?. Journal of Wound Care.

[106] Dunham LJ (1972). Cancer in man at site of prior benign lesion of skin or mucous membrane: a review. Cancer Research.

[107] Freund H, Biran S, Laufer N, Eyal Z (1976). Breast cancer arising in surgical scars. Journal of Surgical Oncology.

[108] Noodleman FR, Pollack SV (1986). Trauma as a possible etiologic factor in basal cell carcinoma. Journal of Dermatologic Surgery and Oncology.

[109] Rigby JE, Morris JA, Lavelle J, Stewart M, Gatrell AC (2002). Can physical trauma cause breast cancer?. European Journal of Cancer Prevention.

[110] Rybinski B, Franco-Barraza J, Cukierman E (2014). The wound healing, chronic fibrosis, and cancer progression triad. Physiological Genomics.

[111] Wang Q, Fiel MI, Blank S, Luan W, Kadri H, Kim KW, Manizate F, Rosenblatt AG, Labow DM, Schwartz ME, Hiotis SP (2013). Impact of liver fibrosis on prognosis following liver resection for hepatitis B-associated hepatocellular carcinoma. British Journal of Cancer.

[112] Goder M, Kornhaber R, Bordoni D, Winkler E, Haik J, Tessone A (2016). Cutaneous basal cell carcinoma arising within a keloid scar: a case report. Onco Targets and Therapy.

[113] He Y, Merin MR, Sharon VR, Maverakis E (2011). Eruptive keloids associated with breast cancer: a paraneoplastic phenomenon?. Acta Dermato-Venereologica.

[114] Bedore J, Leask A, Seguin CA (2014). Targeting the extracellular matrix: matricellular proteins regulate cell-extracellular matrix communication within distinct niches of the intervertebral disc. Matrix Biology.

[115] Venning FA, Wullkopf L, Erler JT (2015). Targeting ECM disrupts cancer progression. Frontiers in Oncology.

[116] Lampi MC, Reinhart-King CA (2018). Targeting extracellular matrix stiffness to attenuate disease: from molecular mechanisms to clinical trials. Science Translational Medicine.

[117] DeGregori J (2013). Challenging the axiom: does the occurrence of oncogenic mutations truly limit cancer development with age?. Oncogene.

[118] Taylor MA, Amin JD, Kirschmann DA, Schiemann WP (2011). Lysyl oxidase contributes to mechanotransduction-mediated regulation of transforming growth factor-beta signaling in breast cancer cells. Neoplasia.

[119] Quan, T., & Fisher, G. J. (2015). Role of age-associated alterations of the dermal extracellular matrix microenvironment in human skin aging: a mini-review. Gerontology, 61, 427–34. 10.1159/000371708 PMC4524793 25660807

[120] Zhao XK, Cheng Y, Liang Cheng M, Yu L, Mu M, Li H, Liu Y, Zhang B, Yao Y, Guo H, Wang R, Zhang Q (2016). Focal adhesion kinase regulates fibroblast migration via integrin beta-1 and plays a central role in fibrosis. Scientific Reports.

[121] Duscher D, Maan ZN, Wong VW, Rennert RC, Januszyk M, Rodrigues M, Hu M, Whitmore AJ, Whittam AJ, Longaker MT, Gurtner GC (2014). Mechanotransduction and fibrosis. Journal of Biomechanics.

[122] Platoni K. E. B. E. (2007). Thinking outside the cell.

[123] Ingber DE (2008). Can cancer be reversed by engineering the tumor microenvironment?. Seminars in Cancer Biology.

[124] Weaver VM, Petersen OW, Wang F, Larabell CA, Briand P, Damsky C, Bissell MJ (1997). Reversion of the malignant phenotype of human breast cells in three-dimensional culture and in vivo by integrin blocking antibodies. Journal of Cell Biology.

[125] Matsubara M, Bissell MJ (2016). Inhibitors of Rho kinase (ROCK) signaling revert the malignant phenotype of breast cancer cells in 3D context. Oncotarget.

[126] Mi K, Xing Z (2015). CD44(+)/CD24(−) breast cancer cells exhibit phenotypic reversion in three-dimensional self-assembling peptide RADA16 nanofiber scaffold. International Journal of Nanomedicine.

[127] Segel, M., Neumann, B., Hill, M. F. E., Weber, I. P., Viscomi, C., Zhao, C., Young, A., Agley, C. C., Thompson, A. J., Gonzalez, G. A., Sharma, A., Holmqvist, S., Rowitch, D. H., Franze, K., Franklin, R. J. M., & Chalut, K. J. (2019). Author correction: niche stiffness underlies the ageing of central nervous system progenitor cells. *Nature*. *573*,130–4. 10.1038/s41586-019-1484-9 PMC7025879 31413369

[128] Martincorena I, Fowler JC, Wabik A, Lawson ARJ, Abascal F, Hall MWJ, Cagan A, Murai K, Mahbubani K, Stratton MR, Fitzgerald RC, Handford PA, Campbell PJ, Saeb-Parsy K, Jones PH (2018). Somatic mutant clones colonize the human esophagus with age. Science.

[129] Yizhak K, Aguet F, Kim J, Hess JM, Kubler K, Grimsby J, Frazer R, Zhang H, Haradhvala NJ, Rosebrock D, Livitz D, Li X, Arich-Landkof E, Shoresh N, Stewart C, Segre AV, Branton PA, Polak P, Ardlie KG, Getz G (2019). RNA sequence analysis reveals macroscopic somatic clonal expansion across normal tissues. Science.

[130] Labidi-Galy SI, Papp E, Hallberg D, Niknafs N, Adleff V, Noe M, Bhattacharya R, Novak M, Jones S, Phallen J, Hruban CA, Hirsch MS, Lin DI, Schwartz L, Maire CL, Tille JC, Bowden M, Ayhan A, Wood LD, Scharpf RB, Kurman R, Wang TL, Shih IM, Karchin R, Drapkin R, Velculescu VE (2017). High grade serous ovarian carcinomas originate in the fallopian tube. Nature Communications.

[131] Jia D, Nagaoka Y, Katsumata M, Orsulic S (2018). Inflammation is a key contributor to ovarian cancer cell seeding. Scientific Reports.

[132] Sage AP, Martinez VD, Minatel BC, Pewarchuk ME, Marshall EA, MacAulay GM, Hubaux R, Pearson DD, Goodarzi AA, Dellaire G, Lam WL (2018). Genomics and epigenetics of malignant mesothelioma. High Throughput.

[133] Roushdy-Hammady I, Siegel J, Emri S, Testa JR, Carbone M (2001). Genetic-susceptibility factor and malignant mesothelioma in the Cappadocian region of Turkey. Lancet.

[134] Bianchi C, Giarelli L, Grandi G, Brollo A, Ramani L, Zuch C (1997). Latency periods in asbestos-related mesothelioma of the pleura. European Journal of Cancer Prevention.

[135] Marinaccio A, Binazzi A, Cauzillo G, Cavone D, Zotti RD, Ferrante P, Gennaro V, Gorini G, Menegozzo M, Mensi C, Merler E, Mirabelli D, Montanaro F, Musti M, Pannelli F, Romanelli A, Scarselli A, Tumino R (2007). Analysis of latency time and its determinants in asbestos related malignant mesothelioma cases of the Italian register. European Journal of Cancer.

[136] Antman KH (1986). Asbestos-related malignancy. Critical Reviews in Oncology/Hematology.

[137] Panou V, Gadiraju M, Wolin A, Weipert CM, Skarda E, Husain AN, Patel JD, Rose B, Zhang SR, Weatherly M, Nelakuditi V, Knight Johnson A, Helgeson M, Fischer D, Desai A, Sulai N, Ritterhouse L, Roe OD, Turaga KK, Huo D, Segal J, Kadri S, Li Z, Kindler HL, Churpek JE (2018). Frequency of germline mutations in cancer susceptibility genes in malignant mesothelioma. Journal of Clinical Oncology.

[138] Mossman BT, Churg A (1998). Mechanisms in the pathogenesis of asbestosis and silicosis. American Journal of Respiratory and Critical Care Medicine.

[139] Liu G, Cheresh P, Kamp DW (2013). Molecular basis of asbestos-induced lung disease. Annual Review of Pathology.

[140] Matsuzaki H, Maeda M, Lee S, Nishimura Y, Kumagai-Takei N, Hayashi H, Yamamoto S, Hatayama T, Kojima Y, Tabata R, Kishimoto T, Hiratsuka J, Otsuki T (2012). Asbestos-induced cellular and molecular alteration of immunocompetent cells and their relationship with chronic inflammation and carcinogenesis. Journal of Biomedicine and Biotechnology.

[141] Greaves M (2018). A causal mechanism for childhood acute lymphoblastic leukaemia. Nature Reviews Cancer.

[142] Izzi V, Heljasvaara R, Pihlajaniemi T (2017). Understanding the extracellular matrix in acute myeloid leukemia. Haematologica.

[143] Prewitz Marina C, Seib F Philipp, von Bonin Malte, Friedrichs Jens, Stißel Aline, Niehage Christian, Müller Katrin, Anastassiadis Konstantinos, Waskow Claudia, Hoflack Bernard, Bornhäuser Martin, Werner Carsten (2013). Tightly anchored tissue-mimetic matrices as instructive stem cell microenvironments. Nature Methods.

[144] Sonawane AR, Platig J, Fagny M, Chen CY, Paulson JN, Lopes-Ramos CM, DeMeo DL, Quackenbush J, Glass K, Kuijjer ML (2017). Understanding tissue-specific gene regulation. Cell Reports.

[145] Janmey PA, Wells RG, Assoian RK, McCulloch CA (2013). From tissue mechanics to transcription factors. Differentiation.

[146] Banerji J, Rusconi S, Schaffner W (1981). Expression of a beta-globin gene is enhanced by remote SV40 DNA sequences. Cell.

[147] Herz HM, Hu D, Shilatifard A (2014). Enhancer malfunction in cancer. Molecular Cell.

[148] Spill F, Bakal C, Mak M (2018). Mechanical and systems biology of cancer. Computational and Structural Biotechnology Journal.

[149] Malandrino A, Mak M, Kamm RD, Moeendarbary E (2018). Complex mechanics of the heterogeneous extracellular matrix in cancer. Extreme Mechanics Letters.

